# Regional homogeneity alterations of resting-state functional magnetic resonance imaging of chronic rhinosinusitis with olfactory dysfunction

**DOI:** 10.3389/fnins.2023.1146259

**Published:** 2023-07-27

**Authors:** Zhiqiang Zhang, Ying Wu, Qing Luo, Junhao Tu, Jiahao Li, Jiaxin Xiong, Huiting Lv, Jing Ye

**Affiliations:** ^1^Department of Otorhinolaryngology, Head and Neck Surgery, The First Affiliated Hospital of Nanchang University, Nanchang, Jiangxi, China; ^2^Department of Radiology, The First Affiliated Hospital of Nanchang University, Nanchang, Jiangxi, China

**Keywords:** regional homogeneity, chronic rhinosinusitis, olfactory dysfunction, rest-state functional magnetic resonance imaging, cerebellum

## Abstract

**Objectives:**

The aim of this study was to assess the brain functional changes of patients with chronic rhinosinusitis with olfactory dysfunction (CRSwOD) using regional homogeneity (ReHo) of resting-state functional magnetic resonance imaging (MRI) scans, and to better explain the occurrence and development of olfactory decline in patients with chronic sinusitis provides a new idea for the study of more advanced olfactory therapy modalities.

**Methods:**

A total of 28 CRSwOD patients, 24 patients with CRS without olfactory dysfunction (CRSsOD), and 25 healthy controls (HCs) were recruited. All subjects underwent olfactory testing, clinical and brief psychological assessments, and MRI scans. A two-sided two-sample *t* test with AlphaSim correction (voxel-*p* < 0.001, cluster size >54 voxels) was used to detect differences between CRSwOD, CRSsOD, and HC groups.

**Results:**

Compared with HCs, the ReHo values in traditional olfactory regions (e.g., parahippocampal gyrus (PHG), hippocampal, olfactory cortex) were increased, and ReHo values in the frontal gyrus, middle temporal gyrus, precuneus, and posterior cingulate gyrus were decreased in CRSwOD patients. The ReHo values in the precuneus and posterior cingulate gyrus of CRSwOD patients were negatively correlated with Questionnaire of Olfactory Disorders-Negative Statements (QOD-NS) scores. Compared with CRSsOD patients, the ReHo values in cerebellar regions were increased and those in the inferior temporal gyrus, precuneus, postcentral, and paracentral gyrus were decreased in CRSwOD patients. The receiver operating characteristic (ROC) curve showed that the mean ReHo values significantly differed between the CRSwOD and CRSsOD groups.

**Conclusion:**

Synchronization of regional brain activity in the regions of the secondary olfactory cortex orbitofrontal cortex (OFC), temporal gyrus, precuneus, and cerebellum may be closely related to the development of olfactory dysfunction. Precuneus and posterior cingulate gyrus may be critical brain areas of action for emotional dysfunction in CRSwOD patients.

## 1. Introduction

Chronic rhinosinusitis (CRS) is a serious health problem that affects 5–12% of the general population, Olfactory dysfunction (OD) is considered one of the four major symptoms of CRS, and the remaining three are nasal congestion, purulent nasal discharge, and facial pain (Fokkens et al., 2020). A recent meta-analysis has shown that the overall prevalence of OD in the CRS population is 67% (Kohli et al., 2017). OD not only leads to impaired nutritional status and decreased quality of life, but it may also increase the probability of one’s life being at risk (Croy et al., 2014). Chronic inflammation of the olfactory cleft promotes apoptosis of olfactory sensory neurons, leading to damage to the olfactory epithelium; loss of sensory neurons may also potentially inhibit olfactory neurogenesis, leading to poor recovery of the neuroepithelium. However, none of these processes fully explain how inflammation contributes to anosmia (Ahmed and Rowan, 2020).

Odorant molecules contact the sensory terminals of olfactory receptor neurons in the nasal epithelium. It then transmits *via* olfactory bulb (OB) axonal projections to the cortex and terminates in several areas of the basal frontal and medial temporal lobes, including the piriform cortex, peri and anterior amygdaloid cortex, and rostral entorhinal cortex. These cortical areas receiving direct input from the OB are collectively called “primary olfactory cortices.” The cortical target of the primary olfactory cortex is the secondary olfactory cortex. The secondary olfactory cortex is mainly located in various brain regions of the limbic and frontal cortices, including the additional amygdala subnuclei, orbitofrontal cortex (OFC), hippocampus, parahippocampal gyrus, cingulate thalamus, cortex, striatum hypothalamus, and the mediodorsal thalamus (Lundstrom et al., 2011; Seubert et al., 2013; Fjaeldstad et al., 2017).

Currently, the UPSIT or its shorter version (SIT, B-SIT) and the Sniffin’ Sticks are the most widely used tools to detect the severity of olfactory loss in CRS, but individual differences (e.g., single molecule sensitivity, culture) limit their usefulness; thus, there is still a need to find more methods to assess the severity of olfactory loss (Hsieh et al., 2017). Functional magnetic resonance imaging (fMRI) provides a noninvasive method of assessing olfactory function in an “objective” manner, contributing to a better understanding of the emergence and progression of OD in humans (Han et al., 2019). Previous population-based studies have confirmed that subjects with OD have reduced brain activation to odor stimuli in the olfactory-related regions, including the piriform cortex, amygdala, OFC, insula, and anterior cingulate cortex compared with normosmic people with normal olfaction (Pellegrino et al., 2016; Han et al., 2018a). Furthermore, artificially inducing short-term olfactory loss by occluding the nasal cavity alters brain activity in odor-related processing areas in the piriform cortex and OFC (Wei et al., 2019). Chronic rhinosinusitis with olfactory dysfunction (CRSwOD) is thought to be closely associated with abnormal functional activation of these brain areas. The diversity of patients (in terms of the cause of olfaction loss) included in most of the previous studies focusing on OD limits the detection of specific group differences (Han et al., 2019). Therefore, it is important to seek reliable brain activation patterns as a potential clinical parameter for future application of fMRI in research of olfactory disturbances (Frohner et al., 2019). Recent work has shown that the detectability and reproducibility of olfactory activation in the primary olfactory cortex are at the same level as those of visual activation in the visual cortex (given that visual cortical activation is the ideal benchmark for generating the most robust fMRI data), and has demonstrated the usefulness of fMRI as a reliable imaging tool in clinical research (Lu et al., 2018).

Regional Homogeneity (ReHo) is a method of analyzing brain activity at the voxel level by examining the synchronization between the time sequences of a specific voxel and its neighboring voxels. The computation of ReHo involves using the Kendall consistency coefficient (KCC) to assess the blood oxygen level-dependent (BOLD) time series. ReHo demonstrates excellent reliability in test–retest studies and provides insights into the localized characteristics of cerebral activity (Zuo and Xing, 2014). Higher ReHo values indicate greater coherence and centrality of local brain activity. Typically, ReHo calculations focus on the low-frequency range, with particular sensitivity to cortical activity observed in low frequencies ranging from 0.01 to 0.08 Hz (Song et al., 2014; Lv et al., 2018). Recent fMRI studies have revealed significant differences in the functional activity and connectivity of the brain’s olfactory regions between patients with olfactory disorders caused by different diseases and healthy controls, these diseases include chronic sinusitis, infections, trauma, and neurological disorders (Van Regemorter et al., 2022). Furthermore, fMRI research has also demonstrated the extent of olfactory system impairment in diseases such as Parkinson’s and Alzheimer’s, shedding light on the underlying mechanisms (Lu et al., 2019). However, these studies typically involve patients with olfactory disorders resulting from multiple etiologies, which may impact the assessment of olfactory dysfunction specifically related to chronic sinusitis. Therefore, the objective of this study is to determine whether there are differences in the regional homogeneity (ReHo) of the olfactory regions between patients with chronic sinusitis, with or without nasal polyps.

## 2. Materials and methods

### 2.1. Study population

Patients with CRSwOD and CRSsOD (age range, 18–55 years) were recruited from the Department of Otorhinolaryngology, Head and Neck Surgery, the First Affiliated Hospital of Nanchang University. The included subjects met the following criteria: (1) were aged from 18 to 55 years; (2) had a diagnosis of bilateral chronic sinusitis and bilateral chronic sinusitis combined with olfactory dysfunction in accordance with the European Position Paper on Rhinosinusitis and Nasal Polyps 2020; (3) were right-handed; and (4) possessed the ability to cooperate with olfactory assessment and imaging studies. Mini-mental state examination (MMSE) and the Patient Health Questionnaire-2 (PHQ-2) were used to exclude dementia or major depression. Patients with CRSwOD or CRSsOD underwent a standard ear–nose–throat examination with endoscopy, computerized tomography (CT) of the sinuses, and an olfactory test, and the subjects were interviewed using the SinoNasal Outcome Test-22 (Snot-22) and the Questionnaire of Olfactory Disorders-Negative Statements (QOD-NS). Age-matched healthy controls (HCs) for CRSsOD and CRSwOD patients were recruited from volunteers, including spouses, and they underwent the same examination as CRSwOD or CRSsOD patients, except for CT, Snot-22, and QOD-NS.

The exclusion criteria for all subjects were as follows: (1) preexisting olfactory and gustatory dysfunction; (2) previous head trauma, and history or evidence of allergic rhinitis and history of chronic sinusitis surgery; (3) history of neurological disease or other serious illness; (4) mental disorders; (5) contraindication to MRI scan; and (6) abnormal brain image.

All subjects obtained relevant information through a written informed consent and this study was approved by the Ethics Committee of First Affiliated Hospital of Nanchang University.

### 2.2. Outcome measures

Mini-mental state examination (MMSE): The total score can range between 0 and 30 points; an illiterate total score (≤19 points), primary school education level total score (≤22 points), or junior high school education level total score (≤26) points is considered to indicate a progressive decline in memory or other cognitive function, whereas a total score of 27–30 points is considered normal cognition (Folstein et al., 1975). Patient Health Questionnaire-2 (PHQ-2): The items included “no interest or pleasure in doing things” and “low mood, depression, or hopelessness.” Each item was scored from 0 (“not at all”) to 3 (“almost every day”), and the total score ranged from 0 to 6. A score of 3 is considered to indicate cases with major depressive disorder (Kroenke et al., 2003). The 22-item Sino-Nasal Outcome Test (Snot-22) score was contains 22 items covering 3 dimensions: physical problems, functional limitations, and affective outcomes (Baumann et al., 2008). The Questionnaire of Olfactory Disorders-Negative Statements (QOD-NS) was including 17 negative statements about the degree of olfactory impairment in patients (Mattos et al., 2018). While the Lund–Kennedy endoscopic score (LKES) and sinus CT Lund–Mackay score were used assess the degree of nasal inflammation (Lund and Kennedy, 1995; Bhattacharyya, 2006).

### 2.3. Olfactory assessment

OD was objectively assessed by the Sniffin ‘Stick Test (Burghart Messtechnik, Wedel, Germany), which uses an odor-containing felt-tip pen. The test was used to assess bilateral odor detection thresholds (T), odor discrimination (D), and odor identification (I), each on a scale from 1 to 16. The TDI is a total score of three components. Based on TDI, olfactory function was classified as follows: normal olfaction, score ≥ 30.5; hyposmia, score between 16.5 and 30.5; and olfactory dysfunction, score < 16.5 (Whitcroft et al., 2017).

### 2.4. MRI parameters

MRI was performed using a 3-Tesla MR unit (Discovery MR750; GE Healthcare, Milwaukee, WI). All subjects were required to close their eyes, keep their heads still, stay awake, and relax during the scanning. The rs-fMRI data were obtained using an echo planar imaging sequence (repetition time [TR] = 2,000 ms; echo time [TE] = 30 ms; flip angle = 90°; matrix = 64 × 64; the field of view [FOV] = 220 × 220 mm; 4-mm slice thickness; and 240 time point acquisitions) and a high-resolution anatomic 3-D T1 imaging sequence (TR = 1,900 ms; TE = 2.26 ms; flip angle = 9°; matrix = 240 × 256; FOV = 215 × 230 mm; slice thickness = 1.0 mm; and 176 sagittal slices). Conventional T2-weighted imaging was used to exclude visible brain structural abnormalities.

### 2.5. Rs-fMRI data preprocessing

Grouped rs-fMRI image data from the CRSwOD, CRSsOD, and HC groups were batch-imported and preprocessed using DPARSF 6.0 package^1^ on a MATLAB R2018a-based platform. The steps were as follows: (1) format conversion; (2) removal of the first 10 time points of the images; (3) time correction, in which the time difference generated by the interval layer scans was eliminated to ensure the same start point of the acquired images; (4) head movement correction, performed to align the images, such that two subjects in the CRSwOD and CRSsOD group with excessive head movement (horizontal movement distance >2.5 mm or rotation angle >2.5°) were excluded, and the mean head translation, mean head rotation, and frame-wise displacement were calculated for each group; (5) spatial normalization, in which the data of each subject after head movement correction were aligned to the MNI standard spatial brain template, and each voxel was resampled to a 3 × 3 × 3 mm voxel size; (6) to remove the impact of nuisance linear regression (Friston-24 head movement parameters, white matter, cerebrospinal fluid and global signals) and low-frequency drift, bandpass temporal filtering (frequency band set to 0.01–0.08 Hz) and linear detrending were performed; and (7) ReHo methods was performed for each subject using DPARSF software to measure the similarity of the time series of one voxel to its neighboring voxels within the whole-brain mask by calculation of Kendall’s coefficient of concordance (KCC). The number of voxels within measured cluster is 27. After ReHo methods, spatial smoothing was applied using the Gaussian kernel (full width at half maximum (FWHM), 6 mm) to improve the signal-to-noise ratio.

### 2.6. Statistical analysis

The SPSS v26.0 software (SPSS Inc., Chicago, IL, USA) was used for data analysis. To assess differences with in the age, TDI scores, PHQ-2, MMSE and nasal endoscopy Lund–Kennedy score (LKES) between CRSsOD, CRSwOD and HC groups, We used to perform two-sample *t* test. For sex ratio comparisons, Pearson’s chi-square test was used. Two-sample *t*-tests were used to analyze the disease duration, sinus CT Lund–Mackay score (LMS), Snot-22 and QOD-NS score of the two groups (CRSsOD and CRSwOD). A significant difference was considered to be indicate by *p* < 0.05.

Statistical analysis for RS-fMRI data was performed by DPABI software, a two-sided two-sample *t* test was used to detect differences between the CRSwOD, CRSsOD, and HC groups. The brain areas were considered significant by the AlphaSim correction, voxel-*p* < 0.001, cluster size >54 voxels. We extracted the average ReHo values of these anomaly clusters from the preprocessed resting-state fMRI data, and an independent two-sample *t* test was performed. *p* < 0.05 indicated a statistically significant difference.

The correlations between ReHo value and Snot-22 score, QOD-NS score, in CRSsOD and CRSwOD groups were analyzed by Pearson correlation. ROC curves were used to identify CRSwOD patients according to certain ReHo values of the brain region. The area under the ROC curve (AUC) was calculated to assess diagnostic capability.

## 3. Results

### 3.1. Demographic and clinical data

A total of 77 participants were recruited, including 28 patients with CRSwOD (17 males, 11 females, age range, 18–55 years; mean age, 38.37 years), 24 patients with CRSsOD (14 males, 10 females, age range, 18–55 years; mean age, 37.56 years), and 25 HC (13 males, 12 females, age range, 23–55 years, mean age, 45.94 years). Demographic data for all of the subjects are summarized in Table 1.

**Table 1:** Demographics and clinical data of CRSwOD, CRSsOD, and HC groups.

	CRSsOD (*n* = 24) 1	CRSwOD (*n* = 28) 2	HC (*n* = 25) 3	*p* value		
				1v2	1v3	2v3
Age (mean ± SD)	37.56 ± 13.49	38.37 ± 15.63	45.94 ± 11.69	0.844	0.061	0.073
Gender (M/F)	14/10	17/11	13 /12	0.910	0.844	0.875
Disease duration, years (mean ± SD)	2.05 ± 0.74	2.18 ± 0.61	——	0.491	——	——
TDI scores (mean ± SD)	34.23 ± 2.79	13.84 ± 3.67	35.86 ± 2.44	<0.001	0.724	<0.001
MMSE (mean ± SD)	28.50 ± 1.14	28.57 ± 1.07	28.84 ± 1.03	0.864	0.659	0.842
PHQ-2 scores (mean ± SD)	1.08 ± 0.90	1.01 ± 1.03	0.83 ± 0.92	0.925	0.879	0.847
Snot-22 scores (mean ± SD)	32.50 ± 13.87	36.31 ± 14.92	——	0.348	——	——
QOD-NS scores (mean ± SD)	53.09 ± 10.65	50.35 ± 8.74	——	0.313	——	——
LKES (mean ± SD)	6.66 ± 2.38	8.06 ± 2.04	0	0.027	<0.001	<0.001
LMS (mean ± SD)	14.16 ± 5.76	17.56 ± 4.81	——	0.024	——	——

SD, standard deviation; TDI, Sniffin’ Sticks test (threshold, T; discrimination, D; identification, I); MMSE, Mini-mental state examination; PHQ-2, Patient Health Questionnaire-2; Snot-22 scores, SinoNasal Outcome Test-22 score; QOD-NS, The Questionnaire of Olfactory Disorders-Negative Statements; LKES, nasal endoscopy Lund–Kennedy score; LMS, Sinus computerized tomography (CT) Lund–Mackay score. The *p*-values of the three groups regarding age, MMSE, TDI scores, PHQ-2 score and LKES were obtained by the ANOVA test. The *p*-value for gender distribution in the three groups was obtained by the chi-square test. The *p*-values of disease duration, SNOT-22 score, QOD-NS scores, and LMS were obtained by the two test between CRSsOD and CRSwOD groups.

Age, sex, MMSE, PHQ-2 scores, and LKES did not significantly differ between the three groups. Disease duration, QOD-NS scores, Snot-22 scores, and LMS did not significantly differ between CRSwOD and CRSsOD. TDI scores in patients with CRSwOD were significantly lower than those in patients with CRSsOD (*p* < 0.001) between the three groups.

### 3.2. ReHo methods

**Figure 1:**
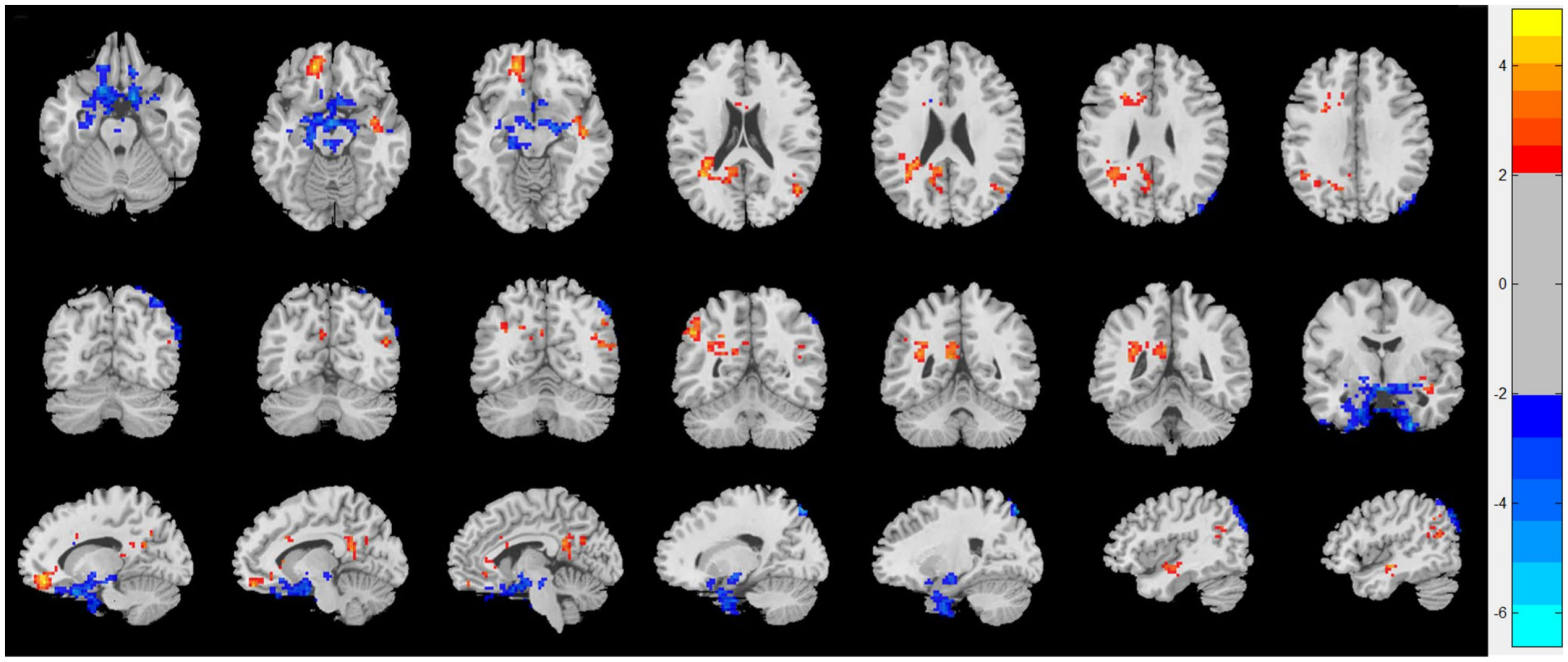
Regional homogeneity (ReHo) changes of the CRSwOD group compared with the HC group (two-sample *t* test; *p* < 0.05, AlphaSim corrected). Warm and cold colors indicate regions with larger and lower ReHo in the former group, respectively.

As shown in Figure 1, compared with HC, patients with CRSwOD showed significantly increased ReHo values in the PHG, hippocampus, fusiform gyrus, olfactory cortex, and angular gyrus; and decreased ReHo values in the precuneus, posterior cingulate gyrus, superior frontal gyrus (medial orbital), gyrus rectus, inferior frontal gyrus (triangular part), superior frontal gyrus (orbital part), and middle temporal gyrus (two-sample *t* test, *p* < 0.05, AlphaSim corrected) (Table 2).

**Table 2:** Differences in ReHo values between CRSwOD, CRSsOD, and HC groups.

Brain areas	Cluster size	L/R	MNI coordinates			T-value
			X	Y	Z	
Differences in ReHo values between CRSsOD and HC						
CRSsOD > HC						
Cerebellum-crus1, cerebellum-6, fusiform gyrus, inferior temporal gyrus	230	L	−42	−42	−36	−2.95
Calcarine fissure and surrounding cortex, cuneus, superior occipital gyrus	177	L	−15	−84	12	3.28
Fusiform gyrus, parahippocampal gyrus	154	L	−24	−3	−39	7.41
CRSsOD < HC						
Frontal-Med-Orb^*^, superior frontal gyrus (dorsolateral), cingulum-Ant^*^	354	L	−18	−9	27	−7.46
Cerebellum-8, cerebellum-9, vermis-8, vermis-9, cerebellum-crus2	350	L	−1	−57	−42	−2.96
Differences in ReHo values between CRSwOD and HC						
CRSwOD > HC						
Parahippocampal gyrus, hippocampus, fusiform gyrus, olfactory cortex	1,100	R	24	−3	−45	7.91
Angular gyrus	81	R	45	−84	24	0.54
CRSwOD < HC						
Precuneus, posterior cingulate gyrus	237	L	−18	−39	15	−7.68
Frontal-Med-Orb^*^, Frontal-Sup-Orb^*^, gyrus rectus, Frontal-Inf-Tri^*^	207	R	12	45	−12	−5.09
Middle temporal gyrus	94	R	48	−63	18	0.54
Differences in ReHo values between CRSsOD and CRSwOD						
CRSsOD > CRSwOD						
Cerebellum-crus1, inferior temporal gyrus	197	L	−33	−63	−39	6.41
Precuneus, postcentral gyrus, paracentral lobule	181	R	12	−27	75	4.73
CRSsOD < CRSwOD						
Cerebellum-9, cerebellum-crus2, and cerebellum-8	226	R	3	−66	−42	−6.85

The coordinates are given as stereotaxic coordinates referring to the atlas of the Montreal Neurological Institute (MNI). cingulum-Ant^*^: anterior cingulate and paracingulate gyri. Frontal-Med-Orb^*^: superior frontal gyrus (medial orbital). Frontal-Inf-Tri^*^, inferior frontal gyrus, triangular part; Frontal-Sup-Orb^*^, superior frontal gyrus, orbital part. Statistical significance was indicated by *p* < 0.05. A two-sided two-sample *t* test with AlphaSim correction (voxel-*p* < 0.001, cluster size > 54 voxels.) was used to detect differences between the CRSwOD, CRSsOD, and HC groups.

**Figure 2:**
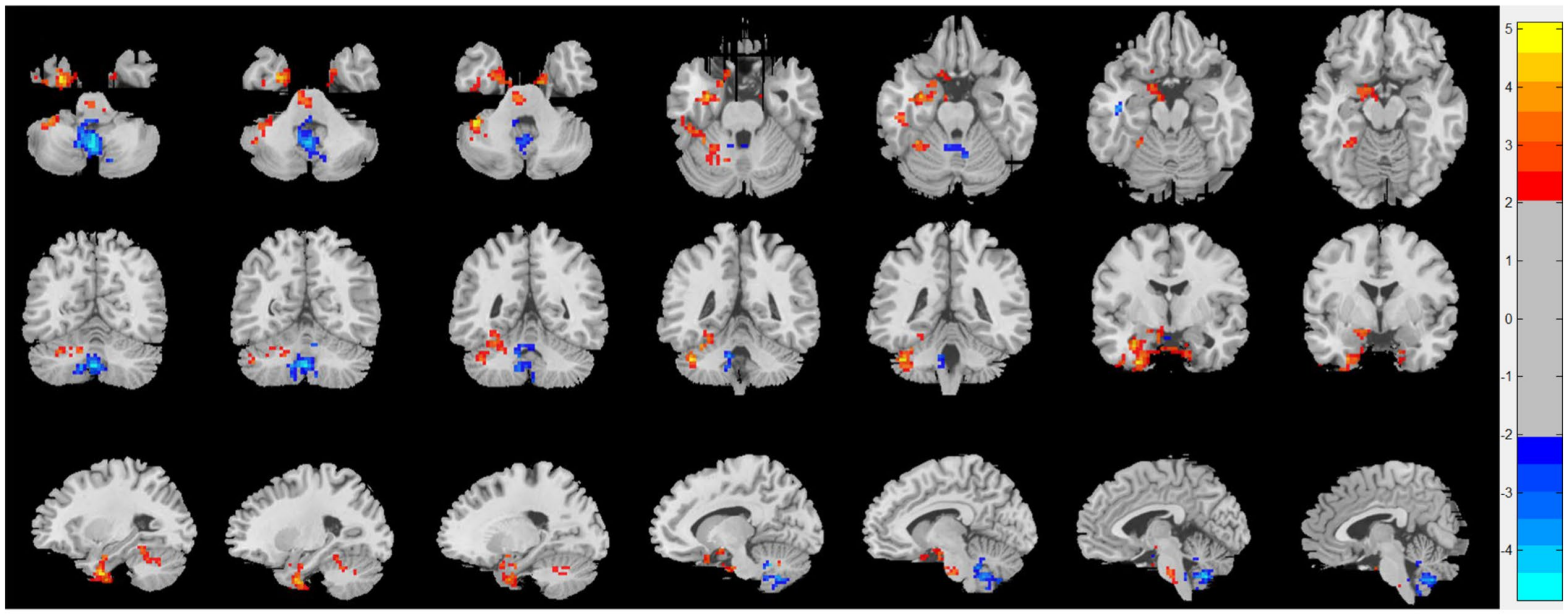
Regional homogeneity (ReHo) changes of the CRSsOD group compared with the HC group (two-sample *t* test; *p* < 0.05, AlphaSim corrected). Warm and cold colors indicate regions with larger and lower ReHo in the former group, respectively.

Compared with HC, patients with CRSsOD showed increased ReHo values in the cerebellum-crus1, cerebellum-6, fusiform gyrus, inferior temporal gyrus, calcarine fissure and surrounding cortex, cuneus, superior occipital gyrus, fusiform gyrus, and PHG; and decreased ReHo values in the superior frontal gyrus (medial orbital), superior frontal gyrus (dorsolateral), anterior cingulate and paracingulate gyri, cerebellum-8, cerebellum-9, vermis-8, vermis-9, and cerebellum-crus2 (two-sample *t* test, *p* < 0.05, AlphaSim corrected; Figure 2; Table 2).

**Figure 3:**
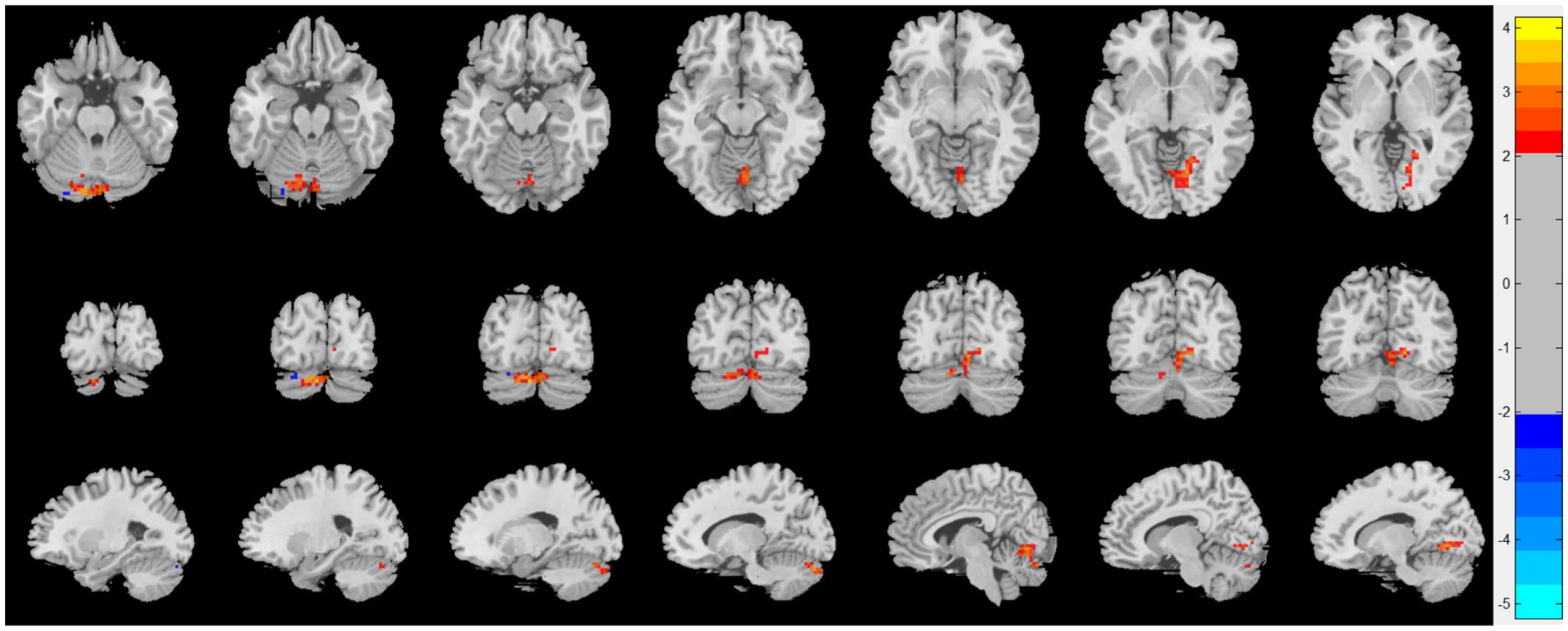
Regional homogeneity (ReHo) changes of the CRSsOD group compared with the CRSwOD group (two-sample *t* test; *p* < 0.05, AlphaSim corrected). Warm and cold colors indicate regions with larger and lower ReHo in the former group, respectively.

Compared with patients with CRSsOD, patients with CRSwOD showed significantly increased ReHo values in the inferior temporal gyrus, precuneus, postcentral gyrus, and paracentral lobule; and decreased ReHo values in the cerebellum-9, cerebellum-crus2, and cerebellum-8 (two-sample *t* test, *p* < 0.05, AlphaSim corrected; Figure 3; Table 2).

### 3.3. Correlation analysis

**Figure 4:**
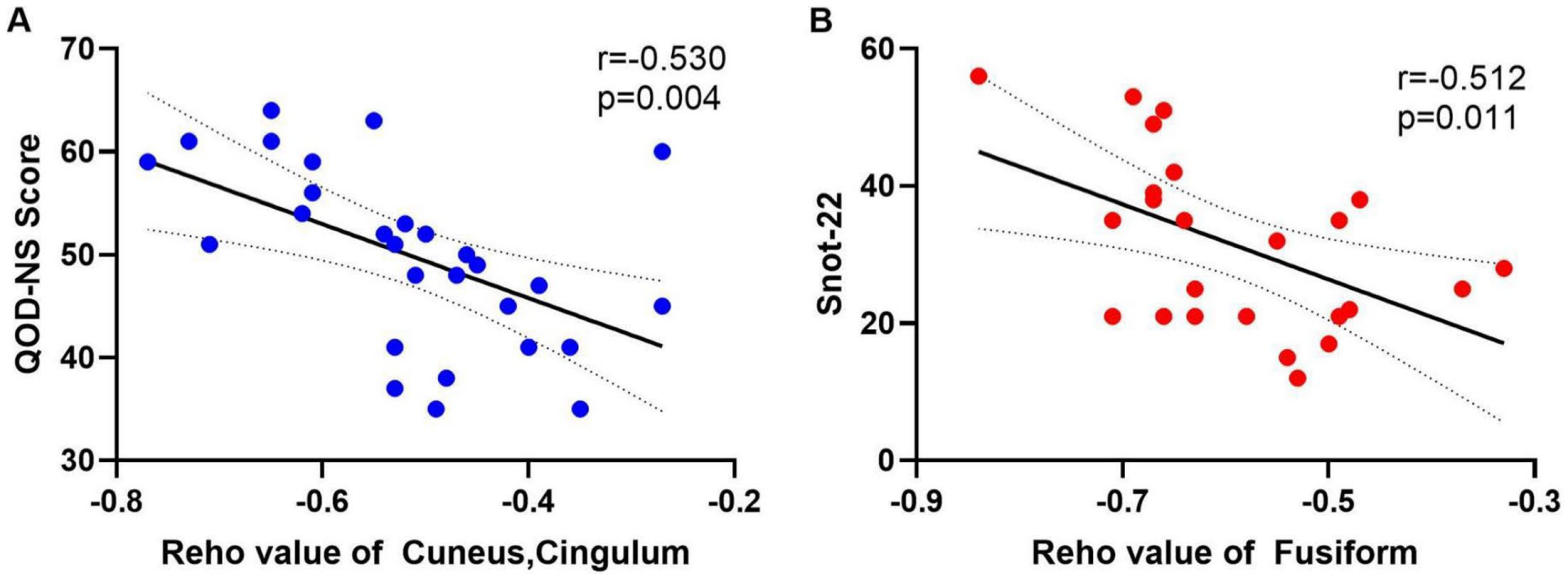
**(A)** The QOD-NS score negatively correlated with the ReHo value of cuneus and cingulum-Ant in CRSwOD group (*n* = 28), *r* = −0.530, *p* < 0.01. **(B)** The SNOT-22 score negatively correlated with the ReHo value of fusiform gyrus in CRSsOD group (*n* = 24), *r* = −0.512, *p* < 0.05.

Snot-22 questionnaire consists of 22 items that cover three dimensions: physiological issues, functional limitations, and emotional outcomes. Each item is scored from 0 to 5, with higher scores indicating poorer health conditions. This instrument is designed to reflect the impact of nasal inflammation on pain, olfaction, and psychological aspects among patients with CRS. QOD-NS questionnaire includes 17 negative statements regarding the extent of olfactory impairment in patients. Participants can agree, partially agree, partially disagree, or disagree with each statement on a scale ranging from 0 to 3. The total score is calculated between 0 and 51, with lower scores indicating a poorer olfactory life experience. We extracted the average ReHo values from the differing brain regions of the CRSsOD and CRSwOD groups, respectively, and conducted Pearson/Spearman correlation analyses with Snot-22 and QOD-NS scores. In patients with CRSwOD, there were significant negative correlations between the QOD-NS scores and ReHo values in the precuneus and posterior cingulate gyrus (*p* < 0.05, *r* = −0.530; Figure 4A), while in patients with CRSsOD, there were significant negative correlations between the Snot-22 scores and ReHo values in the fusiform gyrus (*p* < 0.05, *r* = −0.512; Figure 4B).

### 3.4. ROC curve analysis

**Figure 5:**
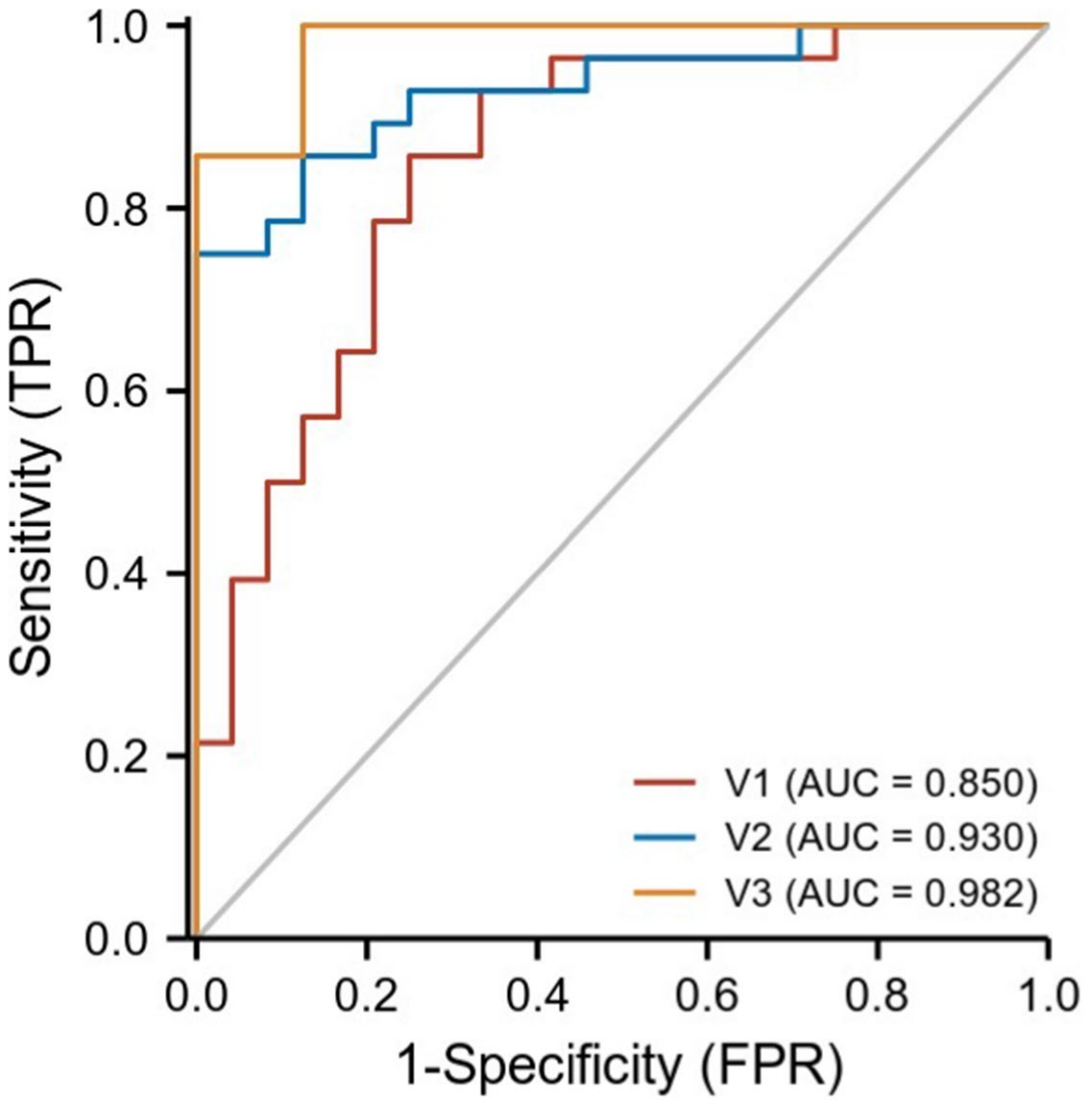
ROC curve analysis of the corresponding brain regions in the identification of CRSsOD and CRSwOD. V1, cerebellum-crus1 and inferior temporal gyrus; V2, precuneus, postcentral, and paracentral gyri; V3, cerebellum-9, cerebellum-crus2, and cerebellum-8.

We hypothesized that ReHo values in certain differential brain regions of CRSwOD and CRSsOD groups could be used as markers to distinguish these groups of patients. We tested this hypothesis by generating receiver operating characteristic (ROC) curves to analyze the responses of specific brain regions that differed significantly in ReHo between the groups. The following regions had a lower AUC for CRSwOD than for CRSsOD: cerebellum-crus1 and inferior temporal gyrus (AUC, 0.850; 95% CI, 0.742–0.957); precuneus, postcentral gyrus, and paracentral lobule (AUC, 0.930; 95% CI, 0.862–0.998). Regions in which AUC was higher in CRSwOD than in CRSsOD were as follows: cerebellum-9, cerebellum-crus2, and cerebellum-8 (AUC, 0.982; 95% CI, 0.957–1.000; Figure 5; Table 3).

**Table 3:** Results of ROC curves in the corresponding brain regions between CRSwOD and CRSsOD.

Brain areas	AUC	95% CI	Cutoff value	Sensitivity	Specificity
Cerebellum-crus1 and inferior temporal gyrus	0.850	0.742–0.957	−0.630	0.857	0.750
Precuneus, postcentral gyrus, paracentral lobule	0.930	0.862–0.998	0.409	0.750	1.000
Cerebellum-9, cerebellum-crus2 and cerebellum-8	0.982	0.957–1.000	−0.417	1.000	0.875

AUC, Area under curve.

## 4. Discussion

FMRI is a reliable tool to assess olfactory function and brain responses to olfactory stimuli in some disease states. Decreased or increased ReHo values indicate that functional activity in certain regions is poorly or highly synchronized compared to controls, further indicating disturbed local regional brain activity, and this approach better reflects the activity status of the whole brain (Zang et al., 2004). In this study, we found differences in brain activity between CRSwOD, CRSsOD, and HC by measuring ReHo values.

Most of the central structures of the olfactory system (entorhinal cortex, piriform cortex, and amygdala) are located in the temporal lobe of the brain. The olfactory center (e.g., the amygdala, olfactory gyrus (entorhinal cortex), anterior olfactory nucleus, and piriform cortex) encompasses the primary olfactory cortical zones (POC), which receive direct olfactory input from the OB and project it to the secondary olfactory structures (SOS) such as the IFG, OFC, hippocampus, PHG, and insula (Zhou et al., 2019). We found significantly different Reho values in the CRSwOD versus HC for traditional olfactory-related areas including piriform cortex, hippocampus, PHG, amygdala, OFC, insula, and anterior cingulate cortex. Our findings are similar to those of functional magnetic resonance imaging studies of the brain in patients with traumatic olfactory dysfunction (Han et al., 2018b; Moon et al., 2018). Compared with HC, the ReHo values of CRSwOD were higher in parahippocampal gyrus, hippocampus, and olfactory cortex, and lower in OFC. This suggests that the POC functional areas of CRSwOD may compensate for the decline in olfactory function by high synchronization of neurons. There was no significant difference in TDI scores between CRSsOD and HC, but ReHo values were lower in OFC. Comparing CRSwOD, CRSsOD, and HC, we found that the ReHo values of temporal gyrus were significantly different; specifically, the ReHo values of temporal gyrus in CRSwOD were lower than those in CRSsOD. Thus, we speculate that the underlying cause of OD may be the poor synchronization of regional brain activity in the OFC and temporal gyrus regions of the secondary olfactory cortex.

The cerebellum, traditionally regarded as a motor structure, is also active in a variety of sensory, affective, and cognitive tasks (Baumann et al., 2015). Although the cerebellum is not considered a part of the olfactory system, cerebellar activity has been reported in olfactory research. Some scholars have found that the cerebellum is insensitive to breathing with low concentrations and comfortable odors, but it can be activated by recognizing high concentrations and unpleasant odors (Sobel et al., 1998; Wabnegger and Schienle, 2019; Zhang et al., 2021). The cerebellum can be divided into an anterior lobe (lobules I through V, parts of lobule VI, and lobule VIII) and a posterior lobe (parts of lobule VI, VII, which includes lobule VIIA in the vermis and crus I and crus II in the hemispheres, lobule VIIB, and most of lobule IX; Stoodley et al., 2012). The anterior part connects to motor cortices, whereas the posterior part is linked with cortical association areas (e.g., prefrontal cortex, posterior parietal cortex, superior temporal regions, cingulate gyrus, and posterior parahippocampal area). These areas are involved in higher-order functions, such as language, working memory, planning, and organizing (Stoodley and Schmahmann, 2009; Schmahmann, 2019). This can explain the low ReHo values in our CRSsOD versus HC comparisons for lobule VIII of cerebellum, lobule IX of cerebellum, and crus II, and in CRSwOD versus CRSsOD comparisons for lobule VIII of cerebellum, lobule IX of cerebellum, and crus II, as higher concentrations and more unpleasant odors are recognized by patients with OD. We hypothesized that the mean ReHo values of certain brain areas may be an effective marker for CRSwOD, and our ROC analysis showed that the AUC of cerebellum-8, cerebellum-9 and cerebellum-crus2 was as high as 0.982. Our results demonstrated that rs-fMRI is a very objective diagnostic tool to overcome the influence of individual differences on olfactory psychophysical test methods. Our results also add evidence for cerebellar involvement in olfaction (Zhang et al., 2021), but the mechanism by which the cerebellum specifically perceives odors needs further investigation.

The brain region of the posterior cingulate gyrus is highly involved in memory–odor associations, which forms long-term memory during adolescence (Arshamian et al., 2013). The posterior cingulate gyrus has been implicated in semantic memory processes, and the strength of its Blood Oxygen on Level Depending (BOLD) response increases with constant rehearsal of situational details to help create more vivid memories (Binder et al., 2009; Bird et al., 2015). Our results showed that the ReHo values in the posterior cingulate gyrus were lower in CRSwOD than in HC, and we believe that the reason for this result is that long-term odors are not recognized by CRSwOD patients; the lack of memory–odor association further affects odor identification and recognition, and the TDI score results in CRSwOD patients also support our idea. The work of Arshamian et al. (2013) supports the notion that odorant cueing of odor-evoked autobiographical memories (OEAMs) leads to more activity in the medial temporal lobe (MTL) areas (e.g., parahippocampal areas) and areas involved in visual activity (e.g., occipital gyrus and precuneus) relative to word cueing. Our study showed that the ReHo value of precuneus was lower in CRSwOD than in HC, while the ReHo value of precuneus was higher in CRSsOD than in HC, which was diametrically opposite. We speculate that the precuneus, a non-traditional olfactory region, is involved in working memory of olfaction, similar to the PHG, which is involved in working memory and is used as a temporary storage system to input and retrieve information from episodic memory (Axmacher et al., 2008; Luck et al., 2010).

Acquired OD has been shown to alter olfactory-related areas such as piriform cortex and orbitofrontal cortex as well as non-olfactory-related areas (Manan et al., 2022). Our results confirm and extend these findings, as our study also found higher ReHo values in CRSwOD than in HC in the angular gyrus of regions associated with multisensory integration processing. It has been suggested that individuals with acquired anosmia show enhanced dynamic functional connectivity between the angular gyrus and operculum, an area associated with basic taste and trigeminal processing (Veldhuizen et al., 2011), although there is no evidence that anosmia contributes to increased acuity to unisensory visual or auditory stimuli and decreased acuity to unisensory taste (Gagnon et al., 2014). Our angular gyrus results support the notion that taste deficits are reported in CRSwOD patients because of reduced flavor experience and perception of retronasal olfactory function.

Olfactory loss or anosmia is frequently accompanied by emotional dysfunction partly due to overlapping brain regions between olfactory and emotional processing centers, such as the limbic system, including the hippocampus, amygdala, and orbitofrontal cortex. Gray matter volume alterations in hippocampus–amygdala and precuneus networks have been found to be associated with olfactory-specific quality of life (QOL) and depressive symptoms in patients with OD using an independent component approach to gray matter volume (Lee et al., 2022). It is well known that the posterior cingulate gyrus is involved in emotional processing. QOD-NS is a valid and reliable olfactory-specific quality of life assessment tool, including emotional assessment (Liu et al., 2022). We found significant differences in ReHo values of the cuneus and cingulum-Ant between the CRSsOD and CRSwOD groups. Additionally, we conducted a correlation analysis between the ReHo values extracted from these different brain regions and the QOD-NS scores. Our study showed lower ReHo values in the precuneus and posterior cingulate gyrus in CRSwOD than in HC, and there was a negative relationship between ReHo values and QOD-NS in these two regions in Pearson correlation analysis in CRSwOD. This suggests that reduced olfactory input may lead to disturbances in regional brain activity in the precuneus and posterior cingulate gyrus, which are associated with low quality of life and depression (Ma and Zhang, 2021; Zhu et al., 2022).

CRS is a serious health problem, which has a wide range of symptoms, such as sinus symptoms, ear symptoms, sleep changes, and pain. The function of fusiform gyrus is associated with recognition and cognitive pain processing neural pathways, and atypical function and structure of the fusiform gyrus are found in patients with chronic low-back pain, fibromyalgia, and cluster headache (Glass et al., 2011; Yang et al., 2013). Alternatively, in studies of impaired intrinsic functional connectivity between the thalamus and visual cortex in migraine patients without aura, the calcarine cortex is considered a critical brain region in physiological mechanisms, and abnormal feedback projections from the visual network can aggravate migraine pain (Wei et al., 2019). The cerebellum is a highly organized brain region located in the dorsal hindbrain of the brainstem and is thought to be involved in sensorimotor, affective, and cognitive information processing (Mehnert and May, 2019). Sleep disturbance is now thought to be associated with activity in the precentral gyrus and nucleus accumbens, whereas fatigue is associated with activity in the prefrontal cortex and nucleus accumbens (Saltiel and Silvershein, 2015), and insomnia is also associated with gray matter volume changes in the left orbitofrontal gyrus and bilateral precuneus (Tang et al., 2015). Compared with HC, we found significant differences in ReHo values in CRSsOD patients in most brain regions, including fusiform gyrus, calcarine fissure cortex and surrounding frontal cortex, cuneus, superior frontal gyrus, and cerebellar regions. There was a negative linear relationship between ReHo values and Snot-22 score in fusiform gyrus (*r* = −0.512, *p* < 0.05) in CRSsOD patients. These results confirm that CRS causes abnormalities in brain functional activity, and we hypothesize that inflammatory cells (e.g., eosinophils, neutrophils) in the nasal cavity of CRS patients stimulate signaling changes regarding pain, mood, and olfactory nerve pathways. Moreover, head/face pain, sleep disturbance, hyposmia/disorder, and depressive mood symptoms are closely related to ReHo values in related brain regions, and we believe that imaging results can be an objective method for quantifying the degree of symptoms in the future.

This study has several limitations that need to be addressed in future studies. First, there was an insufficient sample size, and increasing the sample size will allow for a tighter and more representative calibration of the results. Second, longitudinal studies are also warranted. In future studies, we need to longitudinally track changes in olfactory regions of the brain in patients with CRSwOD and dynamically observe the effects of CRSsOD on olfactory regions in the brain.

## 5. Conclusion

In this study, we used the rs-fMRI ReHo method to find local regional brain activity disturbances in CRSwOD in traditional olfactory regions and some non-olfactory regions. The synchrony of regional brain activity in the OFC, temporal gyrus, precuneus, and cerebellum of the secondary olfactory cortex plays an important role in odor perception. The precuneus and posterior cingulate gyrus may be critical areas of action for emotional dysfunction in CRSwOD patients. The angular gyrus appears to be an important brain region for taste deficits caused by OD.

## Data availability statement

The original contributions presented in the study are included in the article/supplementary material, further inquiries can be directed to the corresponding author.

## Ethics statement

The studies involving human participants were reviewed and approved by the Ethics Committee of the First Affiliated Hospital of Nanchang University. The patients/participants provided their written informed consent to participate in this study.

## Author contributions

JY and ZZ: conception. ZZ, YW, JX, HL, JT, and JL: interpretation or analysis of data. ZZ and YW: preparation of the manuscript and revision for important intellectual content. JY: supervision. All authors contributed to the article and approved the submitted version.

## Funding

This study was supported by the National Natural Science Foundation of China (grant nos. 81860182 and 81460096), Central Funds Guiding the Local Science and Technology Development (20221ZDG020066), and the Innovation Fund Designated for Graduate Students of Jiangxi Province (grant no. YC2020-S128).

## Conflict of interest

The authors declare that the research was conducted in the absence of any commercial or financial relationships that could be construed as a potential conflict of interest.

## Publisher’s note

All claims expressed in this article are solely those of the authors and do not necessarily represent those of their affiliated organizations, or those of the publisher, the editors and the reviewers. Any product that may be evaluated in this article, or claim that may be made by its manufacturer, is not guaranteed or endorsed by the publisher.

## Footnotes

^1^ http://rfmri.org/DPARSF
